# *Mycobacterium avium* subsp. *paratuberculosis* lipophilic antigen causes Crohn’s disease-type necrotizing colitis in Mice

**DOI:** 10.1186/2193-1801-1-47

**Published:** 2012-11-08

**Authors:** Eiichi Momotani, Hiroshi Ozaki, Masatoshi Hori, Shizuo Yamamoto, Takashi Kuribayashi, Shigetoshi Eda, Masahiro Ikegami

**Affiliations:** 1 Research Area of Pathology and Pathophysiology, National Institute of Animal Health, 3-1-5 Kan-nondai, Tsukuba, 305-0856 Japan; 2 Department of Veterinary Pharmacology Graduate School of Agriculture and Life Sciences, the University of Tokyo, Tokyo, 113-8657 Japan; 3 Laboratories of Immunology, School of Life and Environmental Science, Azabu University, Fuchinobe 1-17-71, Chuo-ku, Sagamihara, Kanagawa, 252-5201 Japan; 4 Center for Wildlife Health, Department of Forestry, Wildlife and Fisheries, the University of Tennessee, Knoxville, Tennessee 37996-1071 USA; 5 Department of Pathology, the Jikei University School of Medicine, Minato-ku, Tokyo, Japan

**Keywords:** Mycobacterium, Paratuberculosis, Crohn’s disease, IBD, Mice, Necrotizing colitis, TNBS

## Abstract

Background: A 2,4,6-trinitrobenzene sulfonic acid (TNBS)-induced murine colitis model was developed to investigate the pathogenesis and to evaluate a method of treating human Crohn’s disease. This experimental model rapidly induces colitis similar to human Crohn’s disease lesion in a reproducible manner. However, natural exposure of the human digestive tract to TNBS is unrealistic. A novel animal model based on realistic data is eagerly anticipated in future research on pathogenesis of CD. Method: We evaluated the potency of *Map* antigen molecules in an effort to develop a novel colitis model using a more realistic source than TNBS. We prepared the *Map* antigen by ethanol extraction and developed a mouse model in a manner similar to that of the well-known TNBS-induced colitis in mice. In the experiment, seven days after subcutaneous (SC) injection of the antigen into normal C57BL/6 mice, the same antigen in 50% ethanol was injected into the colon by the transanal route with a fine cannula. Results: On the fifth day after the transanal injection, histopathological examination revealed full-thickness necrotizing colitis with erosion and ulcers; severe infiltration with neutrophils, lymphocytes, macrophages, and perforation. However, no change was detected with each single *Map*-antigen injection. Conclusion: The present results provide a novel animal model for research on CD and may be the key to clarifying the relationship between CD and *Map*. This is the first evidence that mycobacterium antigen induces necrotizing colitis.

## Background

The number of studies attempting to detect clues to the mystery of Crohn’s disease (CD), a chronic intractable intestinal disease, has recently increased (Lakatos 2009; Momotani et al. 2012; Simmons 2010). Althoug various symptomatic treatments and approaches to diet restriction half of CD patients require surgery within 10 years after diagnosis. The risk of postoperative recurrence is 44 to 55% after 10 years (Peyrin-Biroulet et al. 2010). The globally rising rate of pediatric CD is also a major issue (Benchimol et al. 2011; Jakobsen et al. 2008; Phavichitr et al. 2003). No convincing explanation of the pathogenesis of CD currently exists; however, various environmental factors (e.g., pathogenic or non-pathogenic microbes, lifestyle, hygiene factors, diet, and stress) have been suggested (Economou and Pappas 2008; Glasser and Darfeuille-Michaud 2008; Lakatos 2009; Momotani et al. 2012; Neuman and Nanau 2012). Responsible host genes such as the famous NOD2 and a genetic predisposition to CD have been suspected as well (Economou and Pappas 2008; Glasser and Darfeuille-Michaud 2008; Umeno et al. 2011; Vora et al. 2012). The focus in recent years has been on *Mycobacterium avium* subsp. *paratuberculosis* (*Map*) (Behr and Kapur 2008; Eltholth et al. 2009; Momotani et al. 2012), due to the reported pathological similarities of CD and paratuberculosis (Ptb), accumulating reports of frequent detection of Map IS900 DNA, and much less isolation of *Map* from CD lesions (Abubakar et al. 2008; Chiodini 1989; Feller et al. 2007; Momotani et al. 2012). Detection of live *Map* and Map IS900 DNA in children with early-onset CD has been reported (Kirkwood et al. 2009).

Paratuberculosis is a chronic and progressive granulomatous enteritis that affects livestock and wild animals worldwide (Chiodini et al. 1984; Momotani et al. 2012; Nielsen and Toft 2009; Raizman et al. 2009; Stabel et al. 2009). However, differences between CD and Ptb have been pointed out (Momotani et al. 2012; Van Kruiningen 1999). An additional mystery is the “invisible *Map*” that supposedly grows in CD lesions (Momotani et al. 2012; Pierce 2009; Van Kruiningen 1999). This phenomenon has been explained by isolation of a cell-wall-deficient, spheroplastic form of *Map* from human CD lesions (Wall et al. 1993). However, even immunohistochemical staining for cytoplasmic and cell-wall components of *Map* could not detect *Map* in CD lesions (Kobayashi et al. 1989; Momotani et al. 2012; Pierce 2009; Sartor 2005; Van Kruiningen 1999). In contrast to the hypothesis that *Map* infection causes CD, there are no reports of CD-like Ptb lesions in natural or experimental infection with *Map* (Chiodini 1989; Chiodini et al. 1984). Furthermore, intestinal lesions in Ptb of cynomolgus were very similar to those of bovine but differed histopathologically from human CD (McClure et al. 1987).

An experimental colitis model using mice or rats with haptenizing agent 2,4,6-trinitrobenzene sulfonic acid (TNBS) yields pathological findings similar to those of human CD (Arita et al. 2005; Neurath et al. 2000; te Velde et al. 2006). However, natural exposure of the human digestive tract to TNBS is unrealistic. In contrast, exposure of the human digestive tract to *Map* antigen is realistic (Behr and Kapur 2008; Eltholth et al. 2009; Over et al. 2011), since frequently detected Map IS900 DNA in CD patients (Abubakar et al. 2008; Chiodini 1989; Feller et al. 2007; Momotani et al. 2012) is considered to be evidence of *Map* antigen exposure, rather than *Map* infection. In the present study, we prepared a lipophilic antigen of *Map*, and TNBS in the previous TNBS colitis model (Arita et al. 2005) was replaced with the *Map* antigen. Histopathological evaluation revealed severe necrotizing colitis that is very similar to that in the well-known TNBS colitis model. The present study proposes a new CD model and a novel hypothesis on the pathogenesis of human CD.

## Results

### Clinical findings and gross pathology

**Figure 1:**
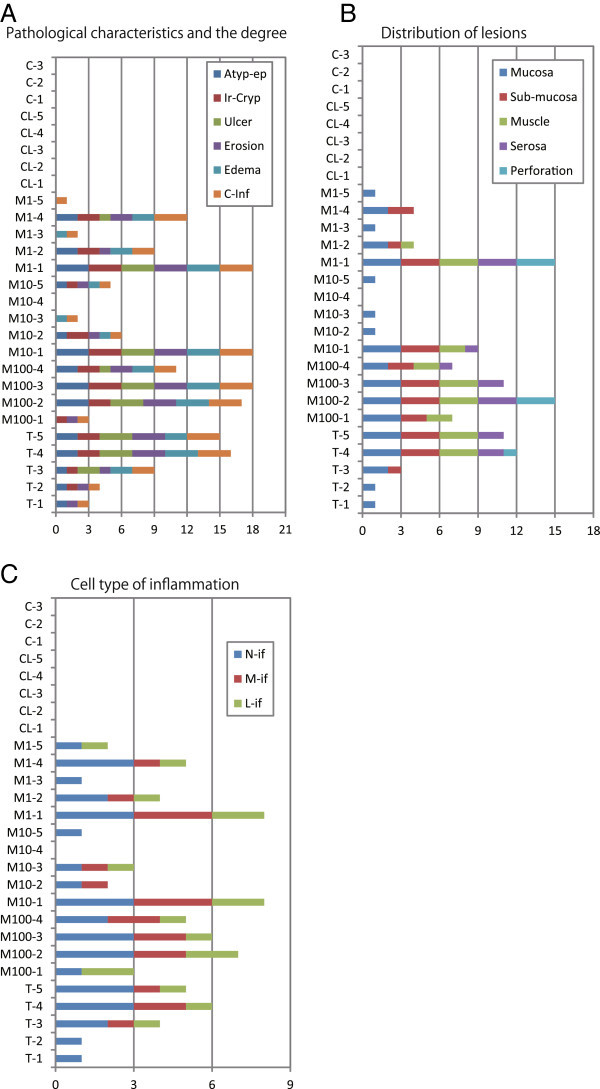
**A. Histopathological data of experimental enteritis generated by TNBS and*Mycobacterium avium*subsp.*paratuberculosis*antigen in C57BL/6 mice.** The longitudinal axis represents types of treatment and numbers of mice. C: Control injected with only vehicle. CL: Mice injected subcutaneously (SC) with vehicle and with MAP-L100 antigen via the intra-colon (IC) route. M1: Mice injected subcutaneously with MAP-l00 plus MAP-L1 via the IC route. M10: Mice injected subcutaneously with MAP-l00 and with MAP-L10 via the IC route. M100: Mice injected subcutaneously with MAP-l00 and with MAP-L100 via the IC route. T: Mice injected subcutaneously with 2.5% TNBS and with 10% via the IC route. Color bars indicate the type of lesion: Atypical epithelial cells (Atyp-ep), irregularly shaped crypt (Ir-Cryp), ulcer (Ulcer), erosion (Erosion), edema (Edema), and cellular infiltration (C-inf). The length of each color bar indicates the intensity (index 0 to 3). The total length of the color bar (horizontal axis) indicates the total degree of enteritis. **B**. Distribution of lesions in lamina propria mucosa (Mucosa), sub-mucosa, muscle layer (Muscle), serosa, and perforation. The degree of lesion (index 0 to 3) in each layer is denoted by the length of the bar. **C**. The type of inflammatory cell (neutrophil (N-if), macrophage (M-if), or lymphocyte (L-if)) is denoted by color, and the degree is denoted by the length of the bars.

During the experiment period, only a few mice exhibited inactivation and a rough coat. During the autopsy, thickening of the colon wall with congestion was observed in the 6 cases exhibited total score than 15 (Figure 1A).

### Histopathology

Stacked bar graphs present histopathological findings regarding degree (Figure 1B), distribution of lesions (Figure 1B), and types of infiltrating cells (Figure 1C). All sections were stained with hematoxylin and eosin (H&E), and the magnification of photos is indicated as a bar.

#### Group 1

**Figure 2:**
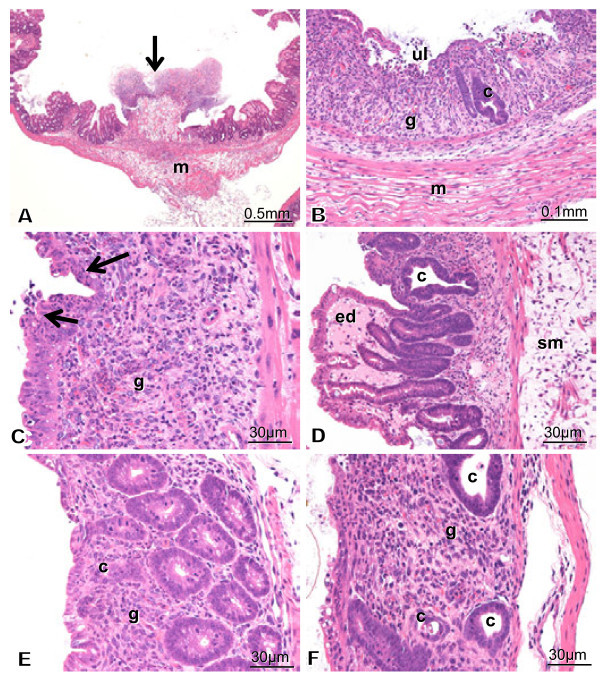
**Histopathology of group 1.** Case T-4 exhibits very severe full-thickness necrotizing enteritis (Figures 2A-D). The most severely damaged tissue is shaped like an erupting volcano (Figure 2A, arrow). Other areas are irregularly shaped. Erosion, ulceration, and granulation (g) are seen (Figures 2**B** and **C**). Intestinal epithelial cells and crypt(c) structure disappear or are modified by inflammation and organization (Figures 2B-D). Neutrophils are the predominant infiltrating cells (Figure 2C). Edema of the muscle layer (m) is observed (Figures 2**A** and **B**). Medium infiltration and edema in the lamina propria mucosa and sub-mucosa (sm) are observed to vary by area (Figure 2D). Erosion and ulceration are seen in T2 (Figure 2E) and 3 (Figure 2F). Crypt (c) structure is irregularly shaped or disappears due to inflammation and granulation (g) (Figures 2E and F).

**Figure 3:**
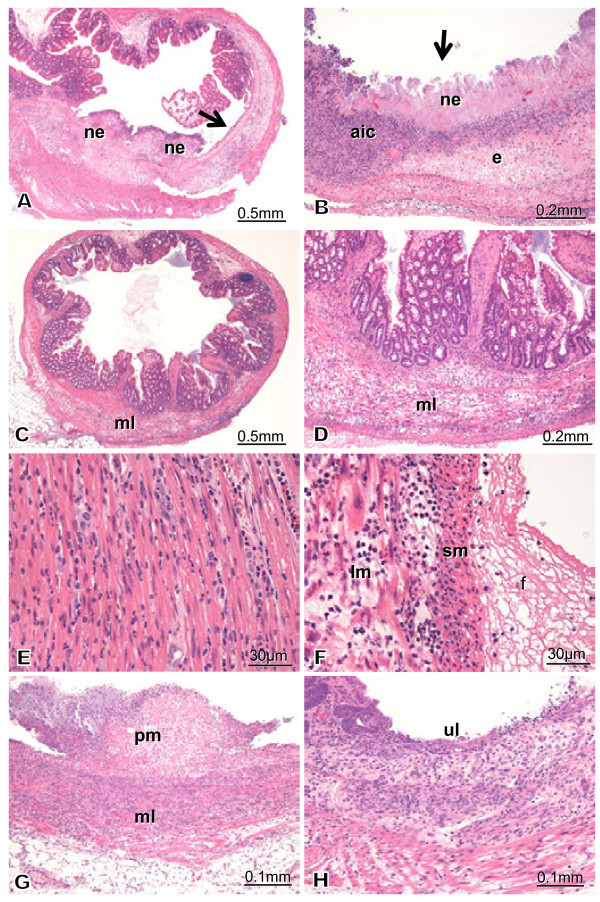
**Histopathology of group 2.** Figures 3**A**-**F** are from case M100-2. Figures 3**G** and **H** are from case M100-3. All sections were stained with hematoxylin and eosin (H&E), and the magnification is denoted by a bar. Case M100-2 exhibited severe full-thickness destructive enteritis (Figures 3A-F). The most severe change was deep ulceration and necrotizing enteritis (ne) (Figures 3A and B). Accumulation of inflammatory cells (aic) and debris, and edema (e) are seen as a pseudomembrane (Figures 3**A** and **B**). The normal structure of the mucosa completely disappeared (arrows); however, the severity differed from area to area in the same case. The structure of the epithelium was maintained in some areas of the colon, but infiltration and edema were observed in the muscle layer (ml) (Figures 3**C** and **D**). Cellular infiltration was observed between circular muscular fibers (Figure 3E). Edema and cellular infiltration of the longitudinal muscle (lm) and serosal membrane occurred (Figure 3F). Fibrin deposition (f) was observed on the serosal membrane (sm) (Figure 3F). Full-thickness necrotizing enteritis is seen in case M100-3 (Figure 3G). Pseudomembrane (pm) is observed (Figure 3F). Infiltration in the muscle layer is significant (Figure 3G). Cellular infiltration and granulation tissue are observed where ulcers (ul) form (Figure 3H).

Histopathological findings caused by TNBS are presented in Figure 2. Three severe (cases T3-5, Figure 2A) and two mild (cases T1 and 2, Figure 2A) colitis cases were observed. Case T-4 exhibited the most severe full-thickness necrotizing colitis (Figure 2A-D). The most severely damaged tissue was shaped like an erupting volcano (Figure 2A, arrow). The colitis included various degrees of erosion, ulceration, and infiltration with neutrophils, lymphocytes, and macrophages in laminapropria mucosa (Figure 2B-F). Atypical epithelial cells including irregularly shaped, vacuolated, and regenerating cells were observed (Figure 2B-F). Epithelium was sometimes infiltrated with neutrophils (Figure 2C, arrows). Granulation (g), an early stage of fibrosis, was observed with ulcer formation (Figures 2B and C). Intestinal epithelial cells and crypt (c) structure on the necrotizing area disappeared or were modified by inflammation and granulation (Figure 2B-F). Neutrophils were the predominant infiltrating cells (Figure 2C); however, other cell types also contributed. Edema (ed) of the muscle layer (m) was observed (Figure 2A and B). Medium infiltration and edema (ed) in the lamina propria mucosa and sub-mucosa (sm) were observed (Figure 2D). Erosion and ulceration were observed in cases T-2 (Figure 2E) and T-3 (Figure 2F).

#### Group 2

Histopathological findings of group 2 are presented in Figure 3. The most severe findings (Figures 3A-F) were observed in case M100-2. Figures 3G and H are from case M100-3. Case M100-2 exhibited severe full-thickness destructive enteritis (Figures 3A-F). The most severe changes were deep ulcer and necrotizing enteritis (ne) (Figures 3A and B). Accumulation of inflammatory cells (aic), debris, and edema (e) were seen as a pseudomembrane (Figures 3A and B). The normal structure of the mucosa completely disappeared (Figure 3, arrow); however, the severity differed from area to area for the same case (Figures 3A and B). In some areas of the colon, the structure of the epithelium was maintained, but infiltration and edema were characteristically observed in the muscle layer (ml) (Figures 3C and D). Cellular infiltration is observed between circular muscular fibers (Figure 3E). Edema and cellular infiltration of the longitudinal muscle (lm) and the serosal membrane (Figures 3D and F) were observed. Fibrin deposition (f) was observed on the serosal membrane (sm) (Figure 3F). Full-thickness necrotizing enteritis was observed in case M100-3 (Figure 3G). The pseudomembrane (pm) was also observed (Figure 3F). Infiltration in the muscle layer was severe (Figure 3G). Cellular infiltration and granulation tissue were observed with ulcer (ul) formation (Figure 3H).

#### Group 3

**Figure 4:**
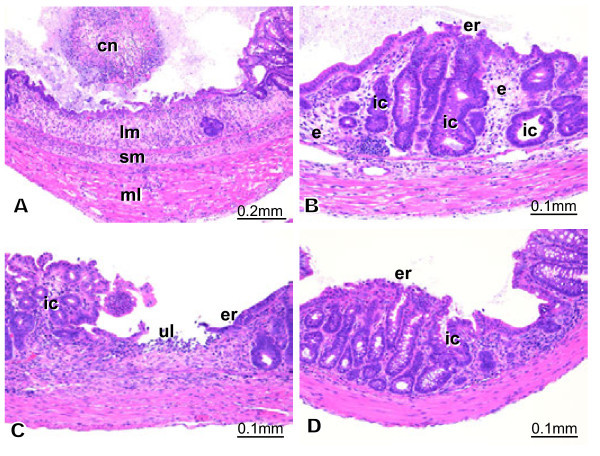
**Histopathology of group 3.** Figures 4**A** and **B** are from case M10-1. Figures 4**C** and **D** are from case M10-2. In M10-1, severe destructive inflammation and development of granulation tissue, and tissue detached by coagulation necrosis (cn) were observed with ulcer formation (Figure 4A). Cellular infiltration was observed mainly in the laminapropria mucosa (lm) and sub-mucosa (sm) but was not severe in the muscular layer (ml) (Figure 4A).Other areas of the colon exhibited erosion (er), edema (e), mild cellular infiltration, and irregular crypt (ic) structures (Figure 4B). Case M10-2 exhibited a small ulcer (ul), erosion (er), cellular infiltration, and irregular crypt (ic); however, no severe necrotizing lesions were apparent.

The histopathology of group3 is presented in Figure 4. Figures 4A and B are from case M10-1. Figures 4C and D are from case M10-2. In M10-1, severe destructive inflammation and development of granulation tissue were observed with ulcer formation (Figure 4A). Cellular infiltration was observed mainly in the laminapropria mucosa (lm) and sub-mucosa (sm) but was not severe in the muscular layer (ml) (Figure 4A). Tissue detached by coagulation necrosis (cn) is depicted in Figure 4A. Other areas of the colon exhibited erosion (er), edema (e), mild cellular infiltration, and irregular crypt (ic) structures (Figure 4B). Case M10-2 exhibited a small ulcer (ul), erosion (er), cellular infiltration, and irregular crypt (ic); however, no severe necrotizing lesions were apparent (Figures 4C and D).

#### Group 4

**Figure 5:**
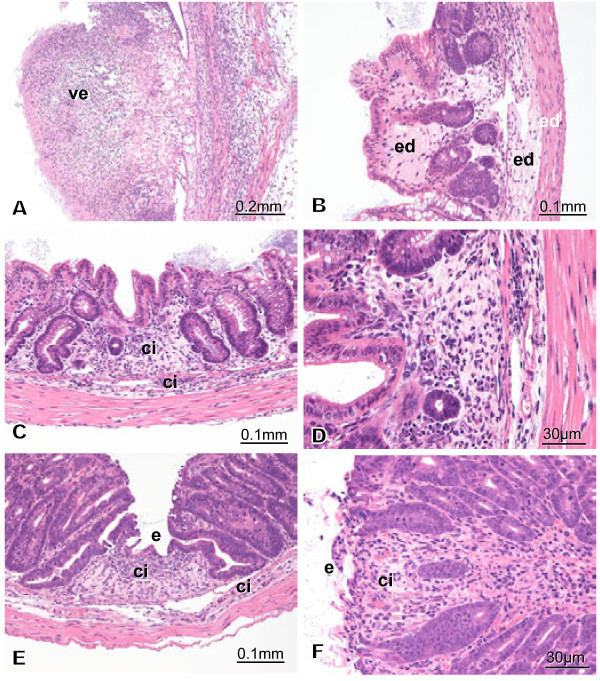
**Histopathology of group 4.** Figure 5**A**-**D** are from case M1-1. Figures 5**E** and **F** are from case M1-4. All sections were stained with H&E, and the magnification is indicated by a bar. Figures 5A indicates very severe full-thickness necrotizing enteritis shaped like an erupting volcano (ve) (Figures 5A). Edema (ed) and cellular infiltration (ci) laminapropria mucosa and sub-mucosa are seen (Figures 5**B** and **C**). The crypt atrophies (Figures 5**C** and **D**). Component cell types are neutrophils, macrophages, and lymphocytes (Figure 5D). Minor erosion (e) and cellular infiltration (ci) are seen in laminapropria mucosa and sub-mucosa (Figures 5E).

The histopathology of the colon section in C57BL/6 mice SC injected with MAP-L100 antigen and then with MAP-L1 via the IC route is depicted in Figures 5A-F. Figures 5A-D are from case M1-1. Figures 5E and F are from case M1-4. Figure 5A indicates very severe full-thickness necrotizing enteritis shaped like an erupting volcano (ve). Edema (ed) and cellular infiltration (ci) of the laminapropria mucosa and sub-mucosa (Figures 5B and C) and atrophy of the crypt were observed (Figures 5C and D). The component cell types of the infiltration are neutrophil, macrophage, and lymphocyte (Figure 5D). Small erosion (e) and cellular infiltration (ci) are seen in the laminapropria mucosa and sub-mucosa (Figures 5E and F).

#### Groups 5 and 6

**Figure 6:**
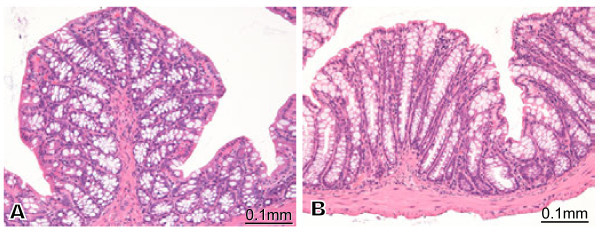
**Histopathology of groups 5 and 6.** There was no lesion in the colon section in C57BL/6 mice (CL-1) injected subcutaneously (SC) with vehicle and then with MAP-L100 via the intra-colon (IC) route (Figure 6A) and CL-1 mice injected SC with vehicle and then via the IC route (Figure 6B).

No lesion was observed in group 5 (Figure 6A) and in group 6 (Figure 6B).

## Discussion

The present study provides the first evidence that the *Map* antigen has the potency to induce colitis that is very similar to mouse TNBS-induced colitis, which has been used as an experimental Crohn’s disease model (Arita et al. 2005; Neurath et al. 2000; te Velde et al. 2006). The nature of the inflammation observed in the present study was very different from that of natural Ptb inflammation (Chiodini et al. 1984; Veazey et al. 1995), but rather similar to human Crohn’s disease enteritis (Loddenkemper 2009; Momotani et al. 2012). However, granuloma formation, a characteristic finding in CD (Loddenkemper 2009; Momotani et al. 2012), was not observed in Map-L and TNBS-induced acute phase colitis in the present study. This finding coincided with previous findings in the study of acute TNBS colitis (Arita et al. 2005; Neurath et al. 2000; te Velde et al. 2006; Wirtz et al. 2007). The necrotizing colitis consisted of erosion, ulceration, and infiltration with neutrophil, lymphocyte, and macrophage. Development of ulceration and regeneration by granulation tissue was also observed. The damaged tissues were restricted, and the adjacent area indicated normal or mild changes in the present TNBS- and *Map*-induced colitis. The finding that the changes may be somewhat similar to “skip lesions” is important in differentiating CD from UC (Wakefield et al. 1989). Every characteristic change in TNBS-induced colitis was observed in the present *Map* antigen-induced lesions. However, cellular infiltration in the muscular layer in *Map* antigen-induced colitis was more prominent than in TNBS-induced colitis (Arita et al. 2005; Neurath et al. 2000; te Velde et al. 2006). In human CD enteritis, severe cellular infiltration or lymphoid follicle formation in the muscular layer is very common (Baumgart and Sandborn 2007; Loddenkemper 2009; Momotani et al. 2012). This *Map*-induced colitis may be closer to human CD lesions than TNBS-induced colitis.

This is the first objective attempt to produce necrotizing colitis by mycobacterial antigen. The results suggest the novel etiological relationship of *Map* to human CD. Traditional etiological studies on CD and *Map* have been carried out under the hypothesis that *“Map* infection” causes CD (Behr and Kapur 2008; Gill et al. 2011; Hermon-Taylor et al. 2000). The effect of a “*Map* antigen” that can contaminate dairy products (Eltholth et al. 2009; Foods 2010; Hermon-Taylor et al. 2000; Millar et al. 1996; Patel and Shah 2011) and meat (Alonso-Hearn et al. 2009; Gill et al. 2011; Klanicova et al. 2011) from Ptb-infected cattle on human health was not reflected as an etiology of CD (Economou and Pappas 2008; Glasser and Darfeuille-Michaud 2008; Lakatos 2009; Umeno et al. 2011). As many previous studies have indicated, the detection of *Map*-specific DNA IS900 by PCR from human intestine, blood, and feces (Abubakar et al. 2008; Chiodini 1989; Tuci et al. 2011) or by in situ hybridization in intestines (Romero et al. 2005) may be evidence of exposure to the *Map* antigen complex but does not always mean the presence of live *Map*. The present results may solve the problem of meeting a requirement of Koch's postulates in analyzing the relationship between CD and *Map* (Lowe et al. 2008).

The facts that colitis in the present study occurred only with repeated exposure to the *Map* antigen and that similar histopathological characteristics were observed after a second exposure to the *Map* antigen in different concentrations suggest the contribution of an immunological mechanism, presumably a delayed-type hypersensitivity (DTH) reaction (Black 1999; Kobayashi et al. 2001). Similar pathogenesis has been considered in TNBS colitis (te Velde et al. 2006). However, further molecular pathological studies are necessary. Recent reports on *Map-*specific reactive CD4 T cells in CD patients (Olsen et al. 2009) suggest the contribution of a DTH reaction generated in the intestine by the *Map* antigen in the pathogenesis of CD. In addition, recent studies on the importance of CD1-presented mycobacterial lipid antigens in the host immune system (Watanabe et al. 2006) provide direction for future research on the pathogenesis of CD.

Epidemiological studies also suggest that the western diet is related to the incidence of CD (Lakatos 2006). The probability that people having some genetic predisposition will ingest live *Map* in food is minimal, but the chance of exposure by heat-killed *Map* antigen may be very frequent. Although the heating process of dairy products eliminates live *Map* (Stabel 2000; Stabel and Lambertz 2004; Stabel et al. 1997), it may not eliminate hazards to human health. Of course, we should not neglect the possibility of human *Map* infection (Hermon-Taylor 2009; Singh et al. 2010).

## Conclusions

The present study proposed a novel mouse model for CD-like colitis and the ability of *Map* antigen to induce necrotizing colitis, which may be the key to understanding the relationship between CD and *Map*. This model may help clarify the pathogenesis of CD, as well as other diseases with a suspected etiological relationship to *Map* (e.g., irritable bowel syndrome (Scanu et al. 2007), multiple sclerosis (Cossu et al. 2011), and type-1 diabetes mellitus (Paccagnini et al. 2009)). Also, this is the first evidence that mycobacterium antigen induces necrotizing colitis. In addition, the authors recommend that people who may have a genetic predisposition (Economou and Pappas 2008; Economou et al. 2004; Glasser and Darfeuille-Michaud 2008; Henderson et al. 2011; Lakatos 2009; Tsianos et al. 2012; Tuci et al. 2011; Umeno et al. 2011) to CD (i.e., if a relative has CD) should avoid dairy products possibly contaminated with the *Map* antigen (Eltholth et al. 2009; Foods 2010; Hermon-Taylor et al. 2000; Millar et al. 1996; Patel and Shah 2011) because no other measures for preventing CD are known.

## Methods

### Antigen preparation

*Mycobacterium avium* subspecies *paratuberculosis* (ATCC 19698) was grown in Middle brook 7H9 liquid medium (Difco Laboratories, MD, USA) enriched with BBL Middle brook OADC (Becton Dickinson, Tokyo, Japan) and 2mg/L of mycobactin J (Allied Laboratory, MO, USA) for two weeks. Next, 90 ml of the culture was centrifuged at 2,200xg for 20min, the supernatant was removed, and the culture was re-suspended in phosphate buffer saline (PBS). After washing twice with PBS, wet bacilli (1g wet weight) were collected. Surface lipophilic antigen was isolated by a previous method (Speer et al. 2006). The bacilli were suspended in 12ml of 80% ethanol by vortex at room temperature for 1min. The suspension was centrifuged, and the resulting supernatant was collected. The dried material weighed 20mg; thus, the concentration was calculated as 1.7mg/ml in 80% ethanol. The material (Map-L antigen) was dissolved with 50% ethanol and used as MAP-L100 antigen (0.68μg/μl). Antigens serially diluted 10 times were used as MAP-L10 (0.068μg/μl) and MAP-L1 antigen (0.0068μg/μl). Preparation of the culture and isolation of the antigen were carried out in laboratory certified as a biosafety level 2 (BSL2-TS-49) in NIAH.

### Experimental animals

C57BL/6Cr Slc female mice at 10 weeks old were purchased from Japan SLC, Inc., and kept in a specific-pathogen-free (SPF) environment under the conditions described above. The mice were fed *ad libitum* during the experiments.

### Experiment procedure

This study was carried out in strict accordance with the recommendations in the Guide for the Care and Use of Laboratory Animals of the National Institutes of Animal Health. The protocol was approved by the Committee of the Ethics of Animal Experiments of the NIAH (Permit Numbers: 08–118, 09–130 and A10-025), and all efforts were made to minimize suffering. All treatments were performed under general anesthesia with Avertin (2,2,-Tribromoethanol) (Arita et al. 2005). The mice were divided into six groups and injected twice with different antigens (Figure 1A). The primary injection was performed subcutaneously. The secondary antigen injections to the colon were performed through the trans-anal route with a fine urinary catheter (Atom Multipurpose Tube, 1.35mm in diameter, Atom Medical Corporation, Tokyo). Feeding was stopped 24 h before the secondary injection. This experimental procedure was basically accorded to previously reported TNBS colitis model by Arita et al. (Arita et al. 2005), because of the the favorable reproducibility.

Group 1 mice, TNBS positive control group were treated subcutaneously with 2.5% TNBS in 50% ethanol and then injected with 10% TNBS in ethanol by the transanal route. All mice in groups 2, 3, and 4 were subcutaneously injected with 25.5μg/150μl of MAP-L antigen in 50% ethanol (Map-L100 antigen). Seven days after treatment, the same antigen with three different concentrations (2.5 (MAP-L100), 0.25 (MAP-L10), and 0.025μg (MAP-L1)/150μl in 50% ethanol) were injected into the colon. Group 5 mice were pretreated subcutaneously with 50% ethanol, and then 25μg of MAP-L100 antigen per 150μl in 50% ethanol was injected into the colon. Group 6 mice were treated with 50% ethanol by SC injection and then by injection into the colon as a non-antigen control. Five days after the injection, the mice were put down by carbon dioxide gas. Colon tissues were sampled and fixed in 20% buffered formalin solution for histopathological examination. The fixative was gently injected into the lumen of the intestine by syringe with a 21 gauge needle.

All experiment protocols used in this study were approved by the Ethics Review Committee for Animal Experimentation of NIAH (approval Nos. 08–118 and 09–130).

### Histopathology

Tissues fixed for three days were trimmed to be round slices and embedded in paraffin blocks. Sections were cut 4μm thick and stained with hematoxylin and eosin (H&E) and then observed under a microscope. The findings were recorded, and the degree of changes were expressed as 0 to 3 and made stacked bar graphs. (Figures A1-3).

## Abbreviations

CD: Crohn’s disease
UC: Ulcerative colitis
Map: Mycobacterium avium subsp. paratuberculosis
Ptb: Paratuberculosis
TNBS: 2,4,6-trinitrobenzene sulfonic acid
OADC: Oleic Acid-Albumin Fraction V-Dextrose-Catalase enrichment
SPF: Specific-pathogen-free.
